# Functional Biogeography of Ocean Microbes Revealed through Non-Negative Matrix Factorization

**DOI:** 10.1371/journal.pone.0043866

**Published:** 2012-09-18

**Authors:** Xingpeng Jiang, Morgan G. I. Langille, Russell Y. Neches, Marie Elliot, Simon A. Levin, Jonathan A. Eisen, Joshua S. Weitz, Jonathan Dushoff

**Affiliations:** 1 College of Information Science and Technology, Drexel University, Philadelphia, Pennsylvania, United States of America; 2 Department of Biochemistry and Molecular Biology, Dalhousie University, Halifax, Nova Scotia, Canada; 3 Genome Center and Microbiology Graduate Group, University of California Davis, Davis, California, United States of America; 4 Department of Biology and M. G. DeGroote Institute for Infectious Disease Research, McMaster University, Hamilton, Ontario, Canada; 5 Department of Ecology and Evolutionary Biology, Princeton University, Princeton, New Jersey, United States of America; 6 Department of Evolution and Ecology, Department of Medical Microbiology and Immunology, University of California Davis, Davis, California, United States of America; 7 School of Biology and School of Physics, Georgia Institute of Technology, Atlanta, Georgia, United States of America; University of Vienna, Austria

## Abstract

The direct “metagenomic” sequencing of genomic material from complex assemblages of bacteria, archaea, viruses and microeukaryotes has yielded new insights into the structure of microbial communities. For example, analysis of metagenomic data has revealed the existence of previously unknown microbial taxa whose spatial distributions are limited by environmental conditions, ecological competition, and dispersal mechanisms. However, differences in genotypes that might lead biologists to designate two microbes as taxonomically distinct need not necessarily imply differences in ecological function. Hence, there is a growing need for large-scale analysis of the distribution of microbial function across habitats. Here, we present a framework for investigating the biogeography of microbial function by analyzing the distribution of protein families inferred from environmental sequence data across a global collection of sites. We map over 6,000,000 protein sequences from unassembled reads from the Global Ocean Survey dataset to 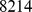 protein families, generating a protein family relative abundance matrix that describes the distribution of each protein family across sites. We then use non-negative matrix factorization (NMF) to approximate these protein family profiles as linear combinations of a small number of ecological components. Each component has a characteristic functional profile and site profile. Our approach identifies common functional signatures within several of the components. We use our method as a filter to estimate functional distance between sites, and find that an NMF-filtered measure of functional distance is more strongly correlated with environmental distance than a comparable PCA-filtered measure. We also find that functional distance is more strongly correlated with environmental distance than with geographic distance, in agreement with prior studies. We identify similar protein functions in several components and suggest that functional co-occurrence across metagenomic samples could lead to future methods for de-novo functional prediction. We conclude by discussing how NMF, and other dimension reduction methods, can help enable a macroscopic functional description of marine ecosystems.

## Introduction

Metagenomics – large-scale sequencing of DNA isolated directly from environmental samples – has greatly facilitated the study of microbial communities [1]–[6]. This wealth of information has created a new set of challenges in understanding the factors underlying the functional processes mediated by microbes at community, regional and global scales [2]. For example, many variants of proteins with similar functions have been identified [7], but little is known about whether such differences have meaningful effects on ecosystem-level function. Further, genome resequencing has revealed that the genetic composition of microbes is highly variable [8]–[12], which suggests that information on taxonomic diversity is insufficient to characterize functional diversity. Thus, a complementary series of analyses are necessary to quantify the functional properties of microbial communities and to explain how differences in their functional properties relate to environmental and geographic factors. Such analyses have the potential to help form the empirical foundation for the study of microbial biogeography [13]–[16].

Data from the Global Ocean Sampling (GOS) [5] expedition has been previously used to investigate the relationship between microbial function and environmental variables. The GOS is appealing for such studies, since it includes samples from diverse locations and habitats, allowing investigation of the interplay among biogeography, environment, and microbial functions. The GOS data set also has important technical advantages, including: numerous samples; consistent and extensive metadata; and long, information-rich, sequence reads. Gianoulis et al [17] introduced a canonical correlation analysis (CCA) framework that was used to identify “metabolic footprints” associated with particular environments. A follow-up study [18] by the same group limited their analysis to 151 membrane protein families and used CCA again to identify relationships between protein families and environmental variables. The most recent functional analysis of GOS uses similar pathway/protein mappings and CCA methods as Gianoulis et al. [17], [18], but incorporates several additional environmental measures [19]. This study found that, of the climatic factors measured, temperature and sunlight were the major determinants of putative biological functions within each sample. Additionally, this study found that environmental, but not geographic, distance between samples was correlated with function.

Eigenvector methods such as CCA and principal component analysis (PCA) are powerful tools for dimensional reduction, but pose problems for biological interpretation, because they represent data by adding and subtracting multiples of components with positive and negative elements, even when the original data has no negative entries (as with functional abundance counts). Here we use non-negative matrix factorization (NMF) [20] methods to gain a complementary perspective on relationships between functions, environment, and biogeography. Using either approach, a community can be represented as a combination of components. NMF approximates samples using components without negative elements, and combines these components by adding positive multiples. In the context of metagenomic profiles, the components represent groups of functional or taxonomic categories that tend to co-occur in samples. Such “parts-based” representations have been useful for the recognition of features in human faces, text and gene expression [20], [21]. In eigenvector-based decompositions, most components have positive sign for some categories and negative sign for others, and samples are also described with positive contributions from some components and negative contributions from others, preventing a straightforward parts-based interpretation.

The lower-dimensional structure identified by NMF methods is often very different from that of eigenvector-based methods. In particular, if microbial communities really are composed of fundamental components that combine in different proportions to make observed communities, NMF will use the data to approximate these underlying “parts”, whereas PCA will find more abstract components which have both positive and negative weights. NMF is an efficient dimension-reduction method that has been previously used in biology, especially in identifying biologically meaningful clusters of co-expressed genes in high dimensional gene expression data sets [21]–[23]. The disadvantage of NMF is that – unlike eigenvector-based methods – it provides only an approximate decomposition, and this decomposition is sensitive to the choice of “rank” – the number of components for NMF factorization.

The starting point for analyses of microbial function biogeography is a matrix containing abundance counts of functional groups or protein families for each of the sites sampled in the study. Previous studies have focused mainly on using the KEGG [24] database since it contains mappings between ortholog groups (KOs) and higher level groupings (KEGG Modules/Pathways), combined with using CCA as their data reduction technique. Here, we instead use the Pfam database [25] which, in addition to full length protein families, contains many shorter protein domain families.

In this study, we make Pfam assignments for over 6,000,000 protein sequences from the GOS, resulting in 8214 unique protein families distributed across 45 sample sites. We then apply an NMF-based framework to investigate patterns of protein family distribution and their correlation with environmental variables. We illustrate how NMF can be used as an effective data reduction method and identify Pfams with common functionality in several of the NMF components. We suggest that future methods could possibly use patterns of co-occurence of protein families across metagenomic samples as a novel non-homology based method for protein function annotation. In addition, we examine the site profiles of the components, and look for associations with geographical and environmental patterns, showing that using NMF as a filter provides further evidence that functional distance correlates better with environmental factors than geographical distance of metagenomic oceanic samples.

## Results

### NMF decomposes high-dimensional data into a small number of components

We selected a subset of 45 out of a total of 79 GOS samples to avoid known problems involving contamination and outliers (see Materials and Methods). Our selection criteria were similar to those used in other studies [17]–[19]. These 45 samples represent a wide geographic and environmental range (Table S7). We then made Pfam assignments for all proteins found in the 45 samples, counted the Pfam assignments for each sample, and normalized these counts by the total number of assignments in the sample (see Materials and Methods). The end result is a “profile matrix” with 45 columns representing the samples and 8214 rows representing Pfams. This profile matrix gives the estimated relative abundance of Pfams in each site and is the starting point for NMF decomposition.

**Figure 1:**
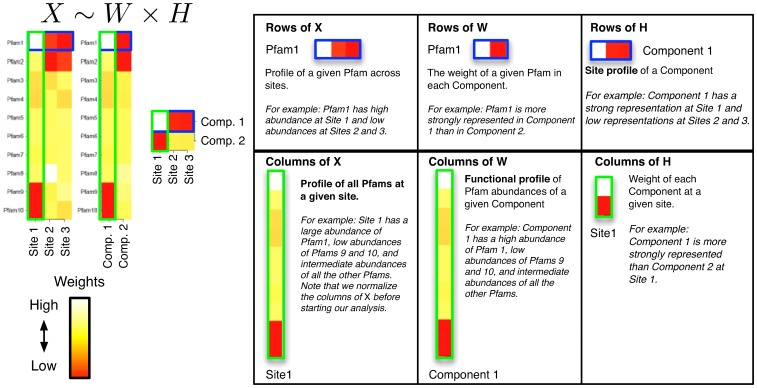
A conceptual illustration of NMF decomposition. Left: We start with a sample of 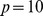 Pfams across 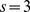 sites, and perform a rank 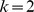 factorization, 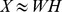. In real applications the reduction in rank is more dramatic. Color codes show Pfam relative abundance. Right: The subfigures illustrate different ways of looking at the decomposition using rows and columns.

The NMF decomposition can be thought of as an empirical attempt to describe observed Pfam patterns in terms of a small number of functional “components” (see Figure 1). Each component is associated with a “functional profile” describing the average relative abundance of each Pfam in the component, and with a “site profile”, describing how strongly the component is represented at each site. Thus, the observed Pfam distribution at a site is approximated as a weighted sum of the functional profiles of our components, with each component's profile weighted by its site profile at that site. In explicit terms, we approximate the observed 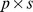 Pfam read matrix (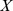) as the product of: a 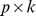 matrix whose 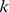 columns are functional profiles for our components (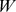); and a 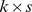 matrix whose 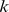 rows are the corresponding site profiles (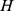). The demonstrative example in Figure 1, uses a factorization of rank 2 (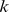) to reduce a 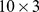 matrix of Pfam abundances (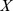) into 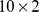 matrix of functional profiles (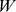) and a 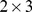 matrix of site profiles (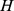). In this example, the best approximation found by NMF has one component with a functional profile very similar to that of the first site, and one that is similar to the remaining two sites.

We applied a concordance method (see Materials and Methods and [26]) to compare possible decomposition ranks, and found that 5 is a suitable rank for the NMF decomposition of the GOS data (Figure 1 in Text S1). This means that the observed Pfam profile matrix (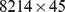) can be stably approximated using 5 functional profiles and associated site profiles.

### Identifying and interpreting the biological basis of functional profiles of components

**Figure 2:**
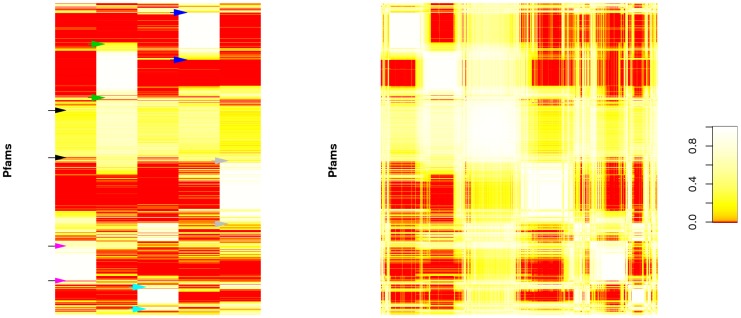
Functional profiles of NMF generated components and the corresponding similarity matrix. a) Five ecological components identified by using NMF across Pfam functional profiles (rows). Colored arrows roughly indicate the clusters of “characteristic” Pfams corresponding to each of the five components; black arrows roughly indicate the cluster of “ubiquitous” Pfams. b) Pfam profile similarity matrices generated using NMF filtering. The matrices are aligned so that the same row corresponds to the same Pfam in each matrix. Pfams with similar profiles are grouped by applying spectral reordering to the similarity matrix (see Materials and Methods). Due to visualization and computational limitations, a random subset of 1000 Pfams are used for ordering and display.

The functional profiles for each of our five components are shown in Figure 2a. Each component has one or more sets of “characteristic” Pfams, which have relatively high abundance within that component and low abundance in other components (See blocks labelled by arrows in Figure 2a). However, unlike some traditional clustering approaches, NMF does not restrict Pfams to be assigned to a single component, and in fact some Pfams are found in high concentration in multiple components. For example, Figure 2a shows areas of overlap occurring on the same row between the large blocks of high concentration near the top of components 2 and 4 (The blocks indicated by blue and green arrows in Figure 2a).

We then constructed a Pfam similarity matrix, using NMF as a *filter* (Figure 2b). This filtered similarity matrix shows clearer patterns of clustering than we find using “direct” similarity or PCA-based filtering (Figure 2 in Text S1). These clusters naturally overlap in many cases, illustrating the advantages of not relying on a strict clustering algorithm. Most of the clusters in the similarity matrix correspond to Pfam blocks dominated by a single component, as can be seen by comparing with Figure 2a. However, the third cluster instead corresponds to Pfams that are broadly distributed across all of the components. This can be seen by comparison with Figure 2a (the Pfam block indicated by black arrows), or by the mid-intensity cross that encompasses the white core of the cluster in in Figure 2b.

**Figure 3:**
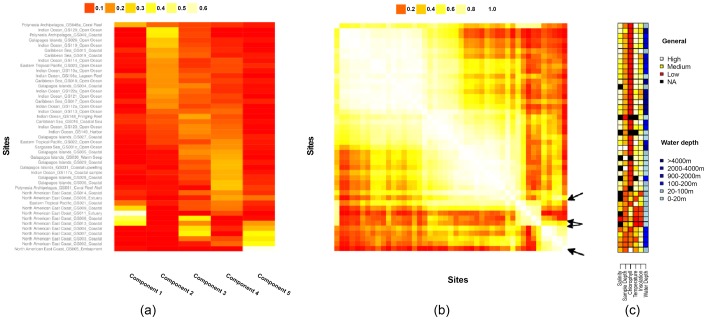
Components across sites. a) Weight for each of the five components at each of the 45 sites (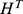); b) the site-similarity matrix (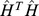); c) environmental variables for the sites. The matrices are aligned so that the same row corresponds to the same site in each matrix. Sites are ordered by applying spectral reordering to the similarity matrix (see Materials and Methods). Rows are aligned across the three matrices.

**Figure 4:**
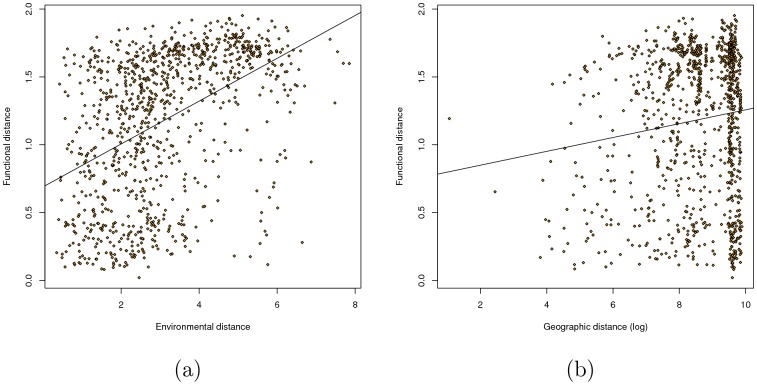
Pairwise correlation between distances. a. Environmental distance vs. functional distance (cor = 0.451, 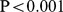, regular Mantel test). b. Logged geographic distance vs. functional distance (cor = 0.127/P = 0.014).

To better understand the functional relevance of the NMF components, we identified Pfams that were strongly associated with each component. We applied NMF on the whole Pfam profile and we selected Pfams based on the *correlation* between their spatial distribution and the site profile of each component (Figure 3a). We contend that this correlation-based approach is preferable to “specificity-” [26] or “projection-” [27], [28] based methods, because it avoids undue bias toward either rare or ubiquitous Pfams (see Materials and Methods and Figure 4 in Text S1).

We found that some of our components had a suite of strongly associated Pfams whose distribution across sites was strongly correlated with the site profile of the component. Component 2 had the clearest group of strongly associated Pfams (126 Pfams have a correlation 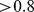 to this component). Components 1 and 5 also had groups of Pfams with relatively high correlation values (28 and 52 Pfams have a correlation 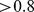 to component 1 and 5 respectively), while Components 3 and 4 did not have any strongly associated Pfams (Figure 7 in Text S1).

To determine if there are particular functions that are associated with each of the components, we manually inspected the lists of Pfams that were most strongly correlated with their respective component. Interestingly, we found commonality in the functional annotation of Pfams associated with components that had strongly associated Pfams (i.e., Components 1, 2, and 5) (Table S1, S2 and S5). Using the 100 most strongly associated Pfams for each component, we found that 40% of the Pfams with known function were related to transport and signalling in Component 1 (which we call “Signalling”) (Table S1); 37% of the Pfams with known function were photosystem-associated in Component 2 (“Photosystem”); and 22% of the Pfams with known function were phage-associated in Component 5 (“Phage”) (See Table S5). In Components 3 and 4, which did not have strongly associated Pfams (Tables S3, S4), we could not identify any functional patterns. Components 3 and 4 may represent combinations of different ecological components that are not separated in this particular decomposition.

The proportion of Pfams without any annotations ranged from 15% (Component 4) to 54% (Component 2: Photosystem). Unidentified Pfams with high association to Components 1, 2 and 5 may have similar functional themes to other Pfams seen in these components, or they may have functions that are ecologically linked to the identified theme, or they may be associated taxonomically rather than functionally (ie., they may be expressed by the same taxa that express the identified Pfams). In the future, we suggest developing statistical methods to identify groups of strong associations, and associated false discovery rates.

Additionally, we inspected the Pfams that were associated with the “ubiquitous” cluster previously identified in Figure 2. Many of these Pfams are associated with bacterial primary metabolism and only 1% of these had unknown functions (Table S6). This is a striking difference compared to the 15–54% proportion of unknown Pfams seen in the five NMF components.

### Characterizing the site profiles of components

Figure 3a shows the estimated site profile for each of the five components. Components 2 (Photosystem) and 4 (Unidentified) are broadly distributed; Components 1 (Signalling) and 5 (Phage) are largely restricted to a handful of sites; and component 3 (Unidentified) shows an intermediate pattern. There is a great deal of overlap between site profiles for different components. For example, component 3 has relatively high similarity to components 2 and 4 (0.57 and 0.65 respectively, see Figure 2 in Text S1 for a similarity heatmap among components).

Figure 3b shows the pattern of filtered similarity between sites. We see clear patterns of grouping, which do not emerge when we calculate functional distances without filtering, or use PCA rather than NMF filtering (Figure 3 in Text S1). As with the Pfams, we see clusters roughly associated with our components, but there is more overlap than with the Pfam clusters (Figure 2b).

Figure 3c shows the distribution of environmental variables measured at each site. Inspection of Figure 3 reveals qualitative correspondence between environmental factors and clusters of similar sites in the similarity matrix. For example, the “North American East Coast” samples are divided into two groups in the bottom right of the similarity matrix (See Figure 3b). Inspection of the environmental features suggests that the split in these samples also corresponds with differences in insolation and water depth.

**Figure 5:**
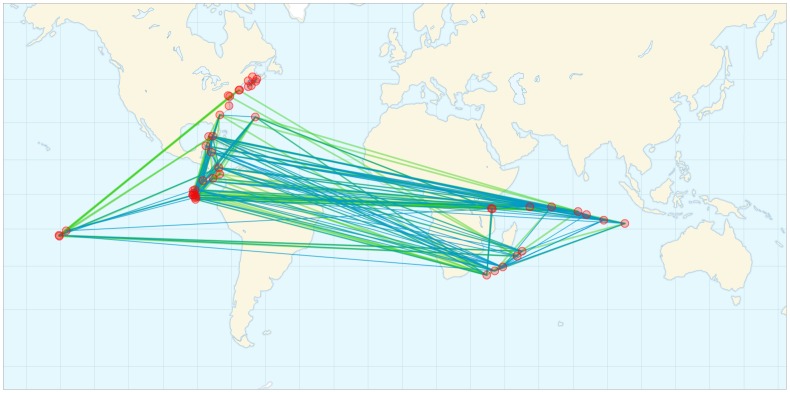
Functional and environmental similarity on a global map. The 120 pairs of sites with highest functional (environmental) similarity are linked in blue (green). Environmental similarity is calculated from the environmental distance matrix 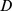 using the transformation 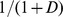. A movie showing this pattern over a range of similarity thresholds is available as Movie S1.

We can also examine patterns of similarity between the components themselves, using site profiles or functional profiles (see Figure 5 in Text S1). All 5 components have strikingly high similarities in their functional profiles, indicating a lot of Pfams which are well represented in many components. Similarity in site profiles is much lower on average, indicating that many pairs of components do not tend to overlap within samples. Overall patterns of similarity also differ: for example, the Phage component (5) and Signalling component (1) have a very high level of functional similarity, but very low similarity in their site profiles.

### Measuring functional distance using an NMF filter

Based on the clear patterns in Figure 3, we hypothesized that NMF-filtered Pfam distance would be a useful metric for functional distance between sites. To test this idea, we compared how well different measures of functional distance were modeled by a combination of environmental distance and geographic distances in a naive regression model. Using adjusted 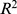 as a measure of overall correlation, we found that the correlation of NMF-filtered Pfam distance with environmental and geographic distances (overall adjusted 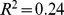) was comparable to that of unfiltered Pfam distance (adjusted 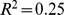), and higher than that of PCA-filtered Pfam distance using the same number of components (adjusted 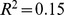). This suggests that the NMF filtering retains more information relevant to these correlations than PCA filtering.

Therefore, we used NMF-filtered Pfam distance to ask about patterns across sites. Specifically, how did functional distance between sites correlate with environmental and geographic distance? Environmental distances were calculated as Euclidean distances of normalized environmental variables (see Materials and Methods), while geographic distances were calculated using great circles. We used logged geographic distances as our main predictor so as not to give too much emphasis to large distances in our linear models.

We found that our measure of functional distance was more correlated with overall environmental distance (Figure 4a) than with logged geographic distance (Figure 4b). We confirmed this result with a multivariate Mantel test; when both distances were used as predictors, the partial correlation between Pfam distance and environmental distance (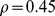, 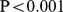) was much higher than that between Pfam distance and logged geographic distance (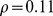, 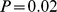). This result was similar to that found by [19], although our partial correlation for environmental distance was substantially higher (0.45 vs. 0.27). Although it was also statistically significant, the partial correlation with geographic distance (0.11) seems so low as to be biologically negligible. These results were robust to different choices of ranks in the NMF decomposition (Figure 8 in Text S1).

Next, we superimposed the Pfam similarity matrix 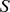 on a global map to visualize how functional differences were influenced by environmental conditions and geographic location (see Figure 5). In the global map we connected sites based on their functional similarity and their environmental similarity respectively. The number of lines connecting sites depended on an arbitrary choice of similarity threshold. A movie showing how this pattern changes over a wide range of thresholds is available as Movie S1. Many early links were established between sites that were well-separated geographically, consistent with our result that the Pfam similarity of microbial communities was more strongly associated with environmental differences than with physical distance.

## Discussion

A significant challenge in metagenomic data analysis is distinguishing important functions and informative patterns from the thousands of functions and/or taxa that are initially identified. In this study, we illustrated how NMF could be used to find functional patterns without supervision. We approximated the GOS dataset of over 6,000,000 unique protein sequences, representing 8214 Pfam abundances distributed across 45 sites, as a combination of five components, each with a characteristic functional profile and site profile. We showed that using this NMF decomposition as a lens allowed identification of novel patterns of clustering of Pfams, and overlaps between these clusters. We looked for groups of Pfams whose distribution across sites was strongly correlated with the identified components, and found three examples of components in which there were identifiable trends in functional annotation corresponding to signalling, photosystem, and phage-associated Pfams.

The NMF lens also allowed us to identify overlapping clusters of the 45 sites in our study. Again, this gave us a novel view on the relationship between sites. In particular, NMF filtering yielded sharper patterns of site similarity than are seen with directly measured similarity or PCA-based similarity (Figure 3 in Text S1). We also found evidence that functional profiles of sites were more strongly correlated with environmental distance than with geographic distance. This correlation has been observed before in the GOS dataset [19], where function was inferred using the KEGG database, rather than the Pfam database used here. In this case, we found that the use of NMF filtering greatly increased the amount of overall correlation seen. This is likely due to filtered distances being less dependent on differences in ubiquitous Pfams. We suggest that filtered distances, and NMF filtering in particular, may provide an improved means to measure the functional distance between sites.

Although we have focused primarily on the use of NMF as a means to analyze function at a community level, NMF may also help to make specific biological predictions in assigning functions to domains of unknown function (DUFs). For example, we found that many Pfams that are strongly associated with component 2 (photosystem-related) and component 5 (phage-related) using our correlation approach (Figure 4 in Text S1) are Domains of Unknown Function (DUFs). Follow-up analysis of the closest taxonomic matches to these DUFs is consistent with many of them sharing function with other members of the component, i.e., DUFs from component 2 have close matches to photoautotrophs (largely cyanobacteria) whereas DUFs from component 5 have close matches to phages.

This clustering of Pfams is similar to the idea of phylogenetic profiling [29], which detects proteins that have similar co-occurrence profiles across hundreds or thousands of genomes and has been used to generate hypotheses for functional annotation of unknown proteins [30]. In contrast, our approach works by associating genes across communities from metagenomic samples. It is important to note that genes associated by this method may be from the same or different organisms; further refinement and testing are needed before any novel annotation can be assigned. For example, it will be of interest to investigate how correlation between DUFs and protein families with known function change as the NMF rank is increased. Moving forward, this sort of “community profiling” could provide a useful tool, which would improve as more metagenomic samples are analyzed.

Metagenomic data provide remarkable detail coding for the functionalities of the species that comprise ecosystems, but much of that detail is likely irrelevant to the robustness of the properties that characterize those systems at macroscopic levels. As we have shown, NMF can help link the microscopic to the macroscopic as part of a statistical framework that extracts the signal from the noise; however more work is needed. Here we have focused on protein families, but deeper understanding of function will require linking these protein families to metabolic pathways. Bridging from metagenomic reads to pathways in broad-scale analyses will allow us to work toward a point where quantitative predictions of community functions can be made based on sequence data as a starting point for detailed biogeochemical analysis. This approach provides hope for developing a macroscopic functional description of marine ecosystems, broadly analogous to so-called “life-zones” in terrestrial ecosystems [31], [32], in which the broad characteristics of ecological communities can be inferred from physico-chemical parameters.

## Materials and Methods

### Datasets

#### Pfam profile

The Global Ocean Sampling expedition [5] is a complex data set. We selected a subset of samples which had been processed in similar ways. In particular, we used only samples with filter size 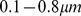, and excluded samples that appeared to represent completely distinct environmental conditions, such as those from freshwater environments. An additional ten samples with very few reads were deleted, while another six samples were excluded due to no hits being found in a preliminary search against the SEED protein database on the MG-RAST server [33]. Lastly, four samples that were extreme outliers in a preliminary NMF analysis (GS000a, GS020, GS032 and GS033) were not included. The final dataset is composed of 45 samples, summarized in Table S7.

A total of 20,729,138 protein sequences from unassembled reads for the 45 samples were downloaded from CAMERA [34], and searched using HMMER 3.0 (http://hmmer.org) against all 11,912 protein families from the Pfam database version 24 [25] using Pfam's per-family gathering threshold cutoffs. The Pfam database has since been updated to version 26, but due to the large computational requirements of the original annotation, version 24 of Pfam was kept for analysis. Multiple Pfams were allowed to be mapped to the same protein since Pfams often represent protein domains and many proteins are multi-domain. In all, 8,040,951 Pfam assignments were identified in 6,010,368 protein sequences and 8214 different Pfams were found at least once in the 45 samples. The number of assignments for each Pfam was counted per sample, and the counts were normalized to the number of Pfams assignments in the sample. The result is a matrix of Pfam relative abundances (Pfam profile matrix) with 8214 rows (one for each Pfam) and 45 columns (one for each sample), whose column sums are equal to one.

#### Geographic distance

Geographic distances were calculated as pairwise distances among sample locations using the great circle route as well as the latitude and longitude recorded in the GOS sample metadata. We used log-transformed geographic distances in correlation analyses so as to not give undue weight to very large distances.

#### Environmental factors

We extracted salinity, sample depth, chlorophyll level, temperature and water depth from the GOS metadata [5], and these values are shown in Table S8. Total incident solar insolation at the surface was obtained from the NASA Surface meteorology and Solar Energy (SSE, http://eosweb.larc.nasa.gov/sse/) Release 6.0 Data Set (Jan 2008) 22-year Monthly 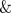 Annual Average (July 1983–June 2005). Missing environmental values were estimated as the average value for the respective variable. We used the square root of water depth in correlation analyses to avoid over-weighting samples taken over the very deep ocean.

### Non-negative matrix factorization (NMF)

If we have 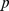 Pfams and 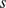 samples, then the size of the profile matrix 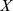 is 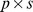. NMF decomposition finds matrices 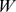 and 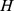, (with dimension 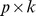 and 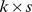, respectively, where 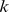 is the *rank* of our factorization) such that 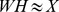. We search for non-negative approximations that minimize the Kullback–Leibler (KL) divergence between 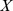 and 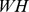 [20], [21].

#### Selecting the rank for NMF decomposition

We have introduced a method based on the 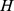 matrix for choosing an appropriate rank (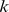) for NMF analysis in the presence of overlap [26]. Approximate factorizations are typically found iteratively from a random starting point [20], and rank is often chosen based on the stability of different realizations of this process. We constructed a symmetric similarity matrix 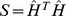, where 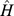 was column-normalized so that 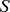 had ones down the diagonal; thus each off-diagonal entry gave the similarity of two samples as seen by our NMF decomposition. We then defined the “concordance index” 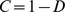, where 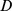 was the mean squared difference between off-diagonal entries of 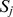 obtained from different realizations of the decomposition [26]. The concordance index 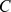 reflected the stability of this matrix across different realizations of the factorization, and was used to select a good decomposition rank 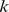.

#### Normalization of 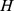 and 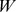


Appropriate normalizations are employed for different purposes. In order to construct sites and Pfams similarity matrices from the results of NMF, we normalize the columns of 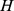 (which are sites) and the rows of 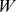 (which are Pfams) respectively so that each similarity matrix has ones down the diagonal.

#### Spectral reordering

To investigate the clustering patterns of samples and Pfams, we employed spectral reordering instead of clustering technology because spectral reordering offers an attractive alternative for clustering [35]. We treated the symmetric, positive, similarity matrix 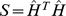 as a weighted graph-adjacency matrix, and applied spectral reordering after an “affinity” transformation [35]. Choosing the scale 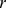 of the affinity transformation is a complex problem [36], [37]. We chose the value of 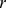 that minimized the Laplacian distance criterion for the untransformed matrix.

### Selecting Pfam similarity groups

We and others [26], [28] have used specificity-based methods (i.e., 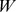-based) to select observed elements similar to NMF basis elements. Specificity-based methods, however, can be sensitive to sampling density (under-sampled Pfams will have a tendency to look specific). Here, therefore, we instead proposed two methods based on similarity and correlation respectively. Given a Pfam 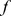 and a component 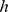, we defined the similarity between them as 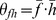, where 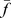 and 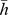 denoted the normalization of them by their Euclidean norms. In the “correlation” method, we used the Pearson correlation coefficient for the correlation between a component profile and a Pfam profile. We found that the correlation method was better than specificity- and similarity-based methods in selecting Pfams. To investigate the possible function of components, we selected the 100 most strongly associated Pfams for each component to investigate their known functions.

### Measuring functional distance between sites

We propose a method for measuring sample distance based on NMF filtering of Pfam profiles. The matrix 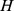 gives the coefficients that approximate each site's functional profile as a linear combination of site profiles. We thus used Euclidean distances between columns of the normalized matrix 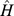 as a measure of functional distance. We called functional distance calculated using 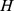 a “filtered” functional distance. We also calculated “unfiltered” distances, based on Euclidean distances between columns of the original Pfam matrix 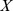.

### Mantel statistics and permutation tests

Mantel tests are used to test the significance of correlations between dissimilarity or distance matrices, while controlling for underlying correlation structure. The statistical method is widely used in ecology studies to test the linear or monotonic independence of the elements in two distance matrices [13], [19]. Furthermore, a recent study suggested that Mantel test is a robust and powerful tool to be used in ecological analysis [38]. The “ecodist” and “vegan” packages in R were used to compute Euclidean distance for the Mantel and partial Mantel statistical analysis. 999 permutations in each test were used to obtain the p-value.

### Pfam function mining

Pfams within the 5 components were manually inspected for possible trends and common functions by looking at the Pfam annotations as well as Gene Ontology annotations using Pfam2GO.

### Scripts and data

All of the data and scripts used in our analysis are available at http://yushan.mcmaster.ca/theobio/GOS_NMF/.

## Supporting Information

Movie S1 **Patterns of functional and environmental similarity visualized on a global map across a range of thresholds.**
(GIF)

Table S1 **Pfams associated with functional component 1 (“Signalling”), along with GO annotations.**
(CSV)

Table S2 **Pfams associated with functional component 2 (“Photosystem”), along with GO annotations.**
(CSV)

Table S3 **Pfams associated with functional component 3 (“Unknown”), along with GO annotations.**
(CSV)

Table S4 **Pfams associated with functional component 4 (“Unknown”), along with GO annotations.**
(CSV)

Table S5 **Pfams associated with functional component 5 (“Bacteriophage”), along with GO annotations.**
(CSV)

Table S6 **Pfams similar to the “ubiquitous” cluster, along with GO annotations.**
(CSV)

Table S7 **Description of selected GOS samples.**
(CSV)

Table S8 **Environmental data associated with selected GOS samples.**
(CSV)

Text S1 **Supporting figures and descriptions.**
(PDF)
