# Supplementary figures and images for: Functional Biogeography of Ocean Microbes Revealed through Non-Negative Matrix Factorization
Source: PLoS One. 2012 Sep 18;7(9):e43866. doi: 10.1371/journal.pone.0043866 (PMC3445553; doi:10.1371/journal.pone.0043866)

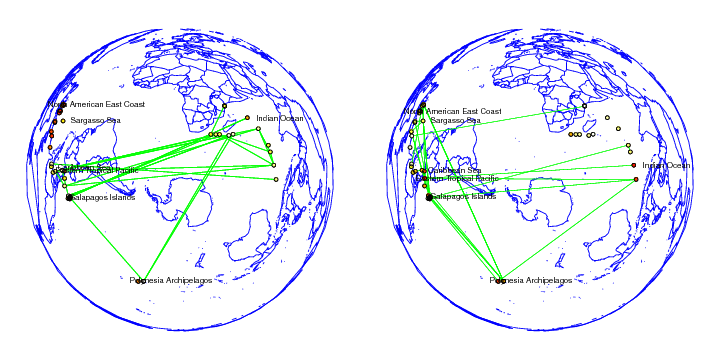

Supplement: Movie S1 — Patterns of functional and environmental similarity visualized on a global map across a range of thresholds. (GIF) [file pone.0043866.s001.gif]
