# Supplementary material for: Functional Biogeography of Ocean Microbes Revealed through Non-Negative Matrix Factorization
Source: PLoS One. 2012 Sep 18;7(9):e43866. doi: 10.1371/journal.pone.0043866 (PMC3445553; doi:10.1371/journal.pone.0043866)
Supplement: Text S1 — Supporting figures and descriptions. (PDF) [file pone.0043866.s010.pdf]

Supplementary Text 1 (Jiang et al.)

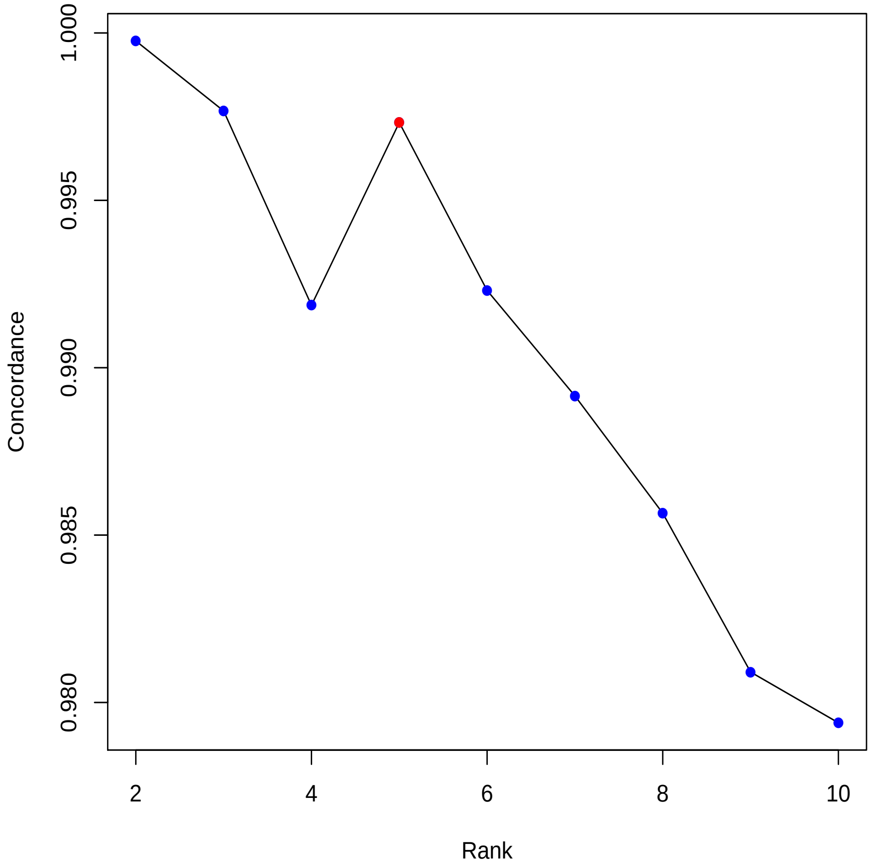

Figure 1: Model selection of NMF. The concordance plot shows the repeatability of realizations of the NMF factorization for different ranks. There are two natural ranks for NMF factorization, given by the two peaks in the plot ($k = 2$ or 5); we used rank 5 for this paper.

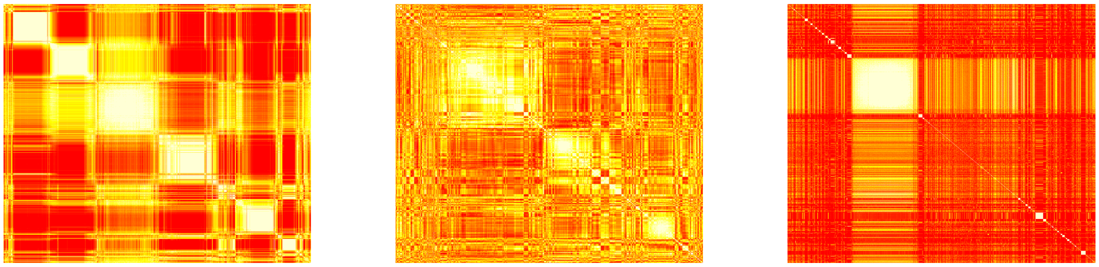

(a) (b) (c)

Figure 2: The comparison of Pfam similarities using NMF, PCA and direct similarity. (a) Pfam similarity using NMF: $\bar{W}\bar{W}^T$, $\bar{W}$ is the row-normalized functional profile matrix; (b) Pfam similarity using the profile matrix reconstructed from the top 5 principal components of PCA; (c) Direct similarity matrix: $\bar{X}^T\bar{X}$, here $\bar{X}$ is the row-normalized Pfam profile matrix.

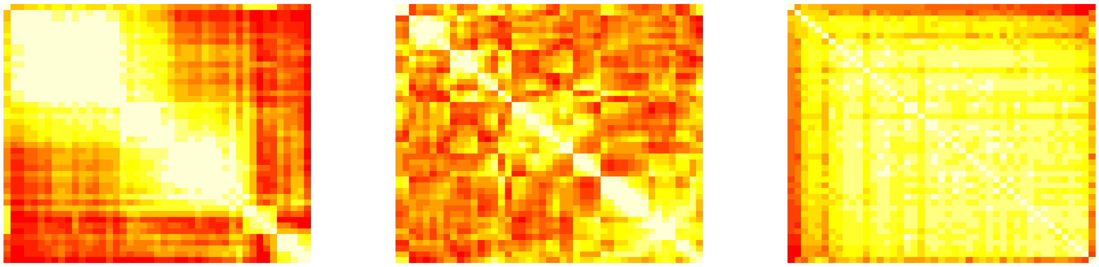

(a) (b) (c)

Figure 3: Comparison of site similarities using NMF, PCA and direct similarity. (a) Site similarity from NMF: $\hat{H}^T\hat{H}$, $\hat{H}$ is the normalized site profiles matrix; (b) Site similarity using the profile matrix reconstructed from the top 5 principal components of PCA; (c) Direct similarity matrix: $\hat{X}^T\hat{X}$, here $\hat{X}$ is the column-normalized Pfam profile matrix.

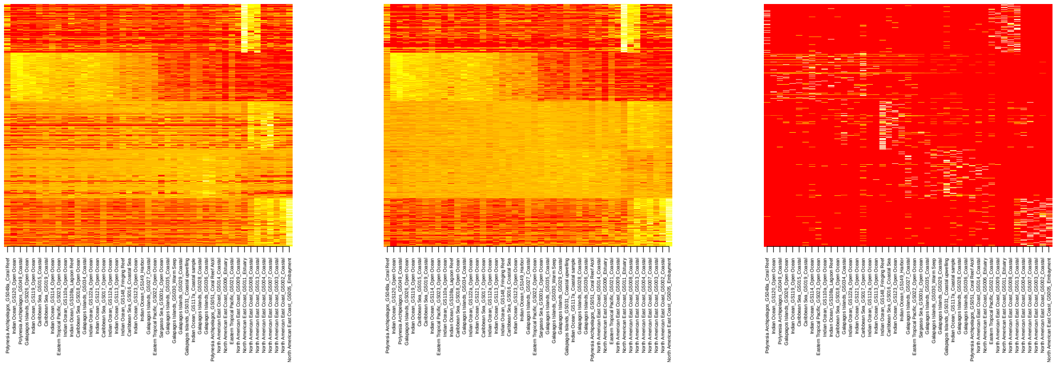

(a) (b) (c)

Figure 4: Comparison of methods for selecting Pfams associated with functional components. 100 pfams are selected for each component based on three methods: (a) correlation; (b) similarity; (c) specificity.

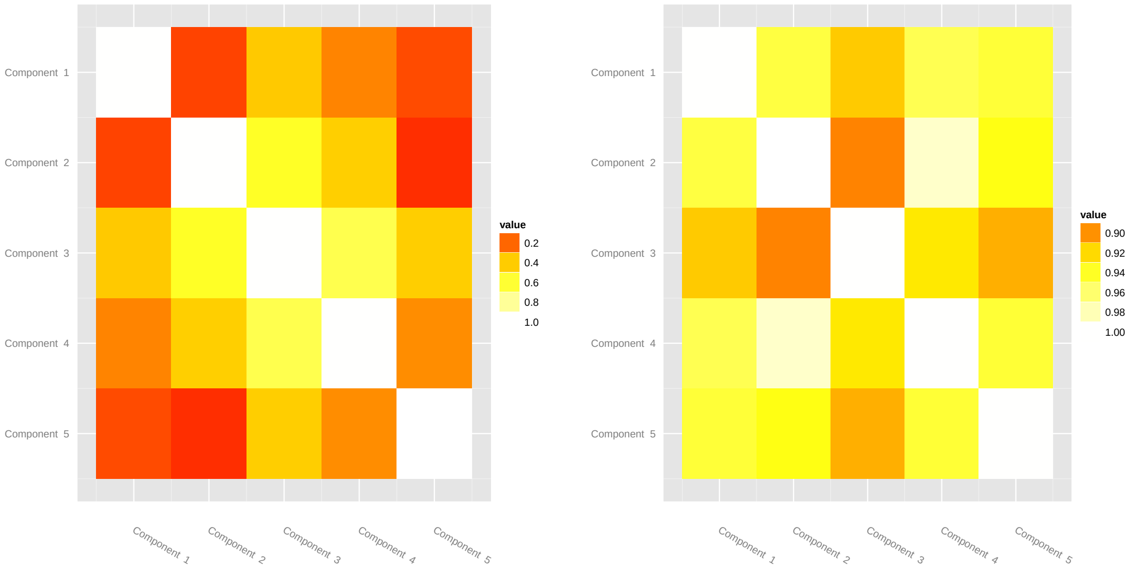

(a) (b)

Figure 5: The similarity among components from (a) site profiles (b) functional profiles.

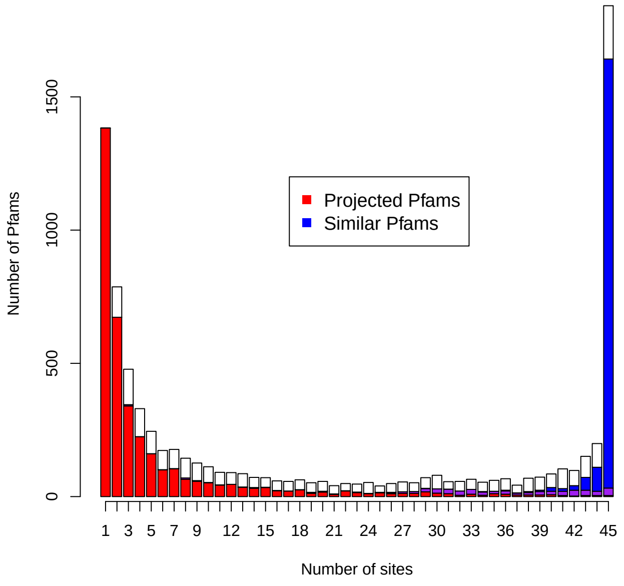

(a)

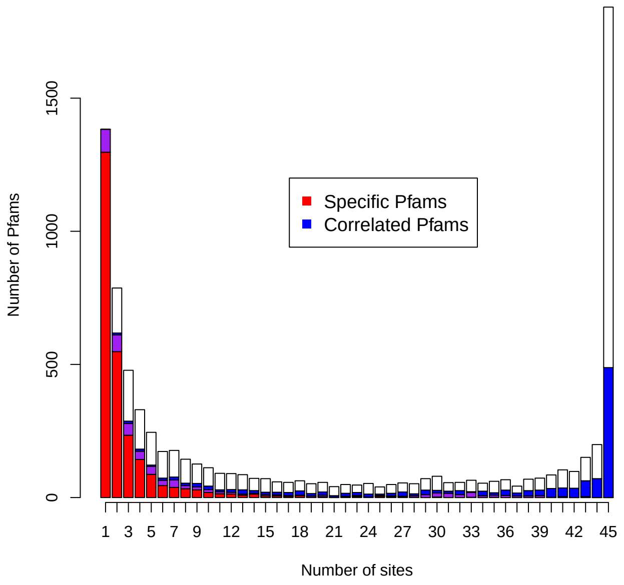

(b)

Figure 6: A comparison of Pfam selection methods. For each selection method, we show the distribution of how many sites selected Pfams are found in. Pfams that are selected by both methods in each panel are shown in purple. Choosing by projection or specificity gives a strong bias towards Pfams found in only a few sites, while choosing based on similarity gives a strong bias towards those found in many sites. Correlation gives a more balanced result.

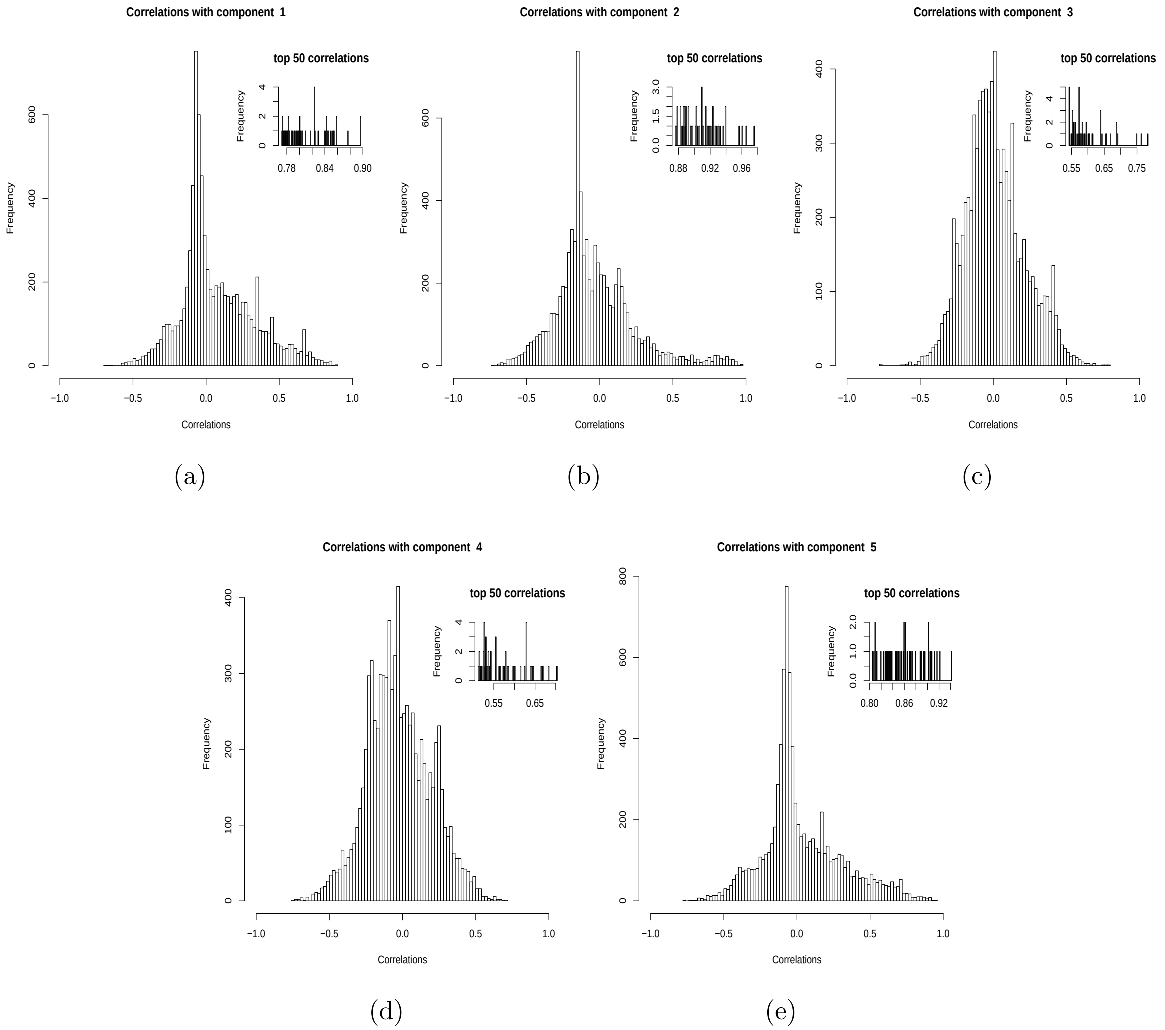

Figure 7: For each component, the distribution of correlation coefficients between Pfam distributions across sites and the site profile associated with the component. These are precisely the values we used to select the top 100 Pfams associated with each component.

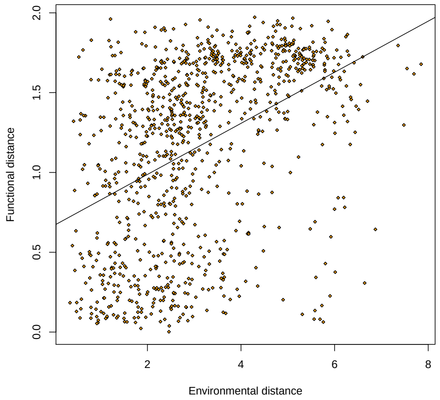

(a)

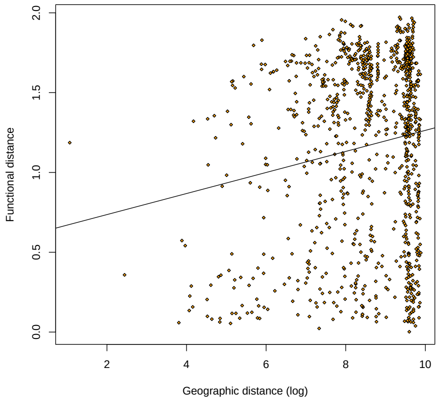

(b)

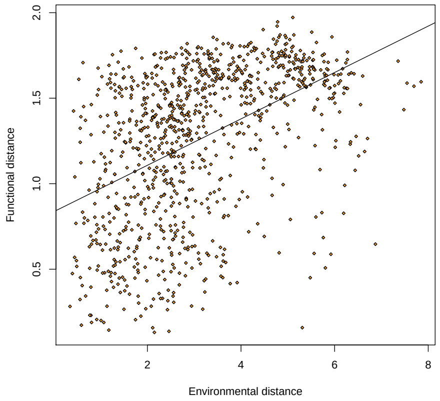

(c)

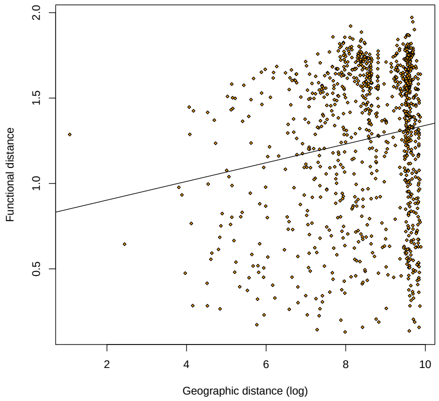

(d)

Figure 8: Biogeographic analysis using rank 4 and rank 6 non-negative factorization. Left: Environmental distance vs. functional distance; The partial correlation coefficients between them conditional to logged geographic distance are 0.424 ($P < 0.001$) and 0.46 ($P < 0.001$) respectively, see (a) and (b). Right: Logged geographic distance vs. functional distance.The partial correlation coefficients between them conditional to environmental distance are 0.137($P < 0.004$) and 0.147 ($P < 0.003$) respectively, see (c) and (d).
